# Dietary Patterns, Frailty and Plasma Proteomic Mediators in the Jackson Heart Study

**DOI:** 10.1093/geroni/igaf122.1871

**Published:** 2025-12-31

**Authors:** Toshiko Tanaka, Luigi Ferrucci, Yichen Jin, Xiyuan Zhang, Katherine Tucker, Sameera Talegawkar

**Affiliations:** National Institute on Aging, Baltimore, Maryland, United States; National Institute on Aging, Baltimore, Maryland, United States; Milken Institute School of Public Health, Washington, District of Columbia, United States; University of Massachusetts Lowell, Lowell, Massachusetts, United States; University of Massachusetts Lowell, Lowell, Massachusetts, United States; Milken Institute School of Public Health, Washington, District of Columbia, United States

## Abstract

Following a balanced and healthful diet is an important modifiable risk factor to slow the rates of aging, however, the mechanisms underlying this association is unclear. We examined this question in the 3645 participants from the Jackson Heart Study. A 34-item frailty index (FI) was constructed that showed association with risk of mortality (HR: 1.48; 95%CI:1.31-1.66). Adherence to four dietary patterns was measured: Mediterranean diet score (MDS), Dietary Approaches to Stop Hypertension (DASH) diet, Healthy Eating Index 2010 (HEI), and Alternate HEI 2010 (AHEI). Greater adherence to DASH (β=-0.063±0.014, p < 0.0001), HEI (β=-0.046±0.015, p = 0.003), or AHEI (β=-0.075±0.014, p < 0.0001) was associated with lower FI while no significant association was observed for MDS (β=-0.022±0.014, p = 0.122). Using plasma proteomic data from a subset of 1,406 participants, we identified associations between dietary patterns and protein levels: 25 proteins linked to the DASH diet, 20 to the AHEI diet, and 1 to the HEI diet. Notably, four proteins (CNTN4, EGFR, KYNU, SEMA6A) were associated with the DASH diet, nine proteins (CHL1, CNTN4, PI3, EGFR, IL1RAP, KYNU, MMP2, MAP2K2, NCAM1) with the AHEI diet, and one protein (EGFR) with the HEI diet. These proteins significantly mediated the relationship between dietary patterns and FI. Among the mediating proteins, six (CNTN4, SEMA6A, CHL1, MMP2, MAP2K2, NCAM1) are known to be involved in axon development and guidance. These findings suggest that adherence to specific dietary patterns influences plasma protein abundances and that the regulation of the nervous system may be a key mechanism linking diet to frailty.

